# The Zebrafish Embryo as a Model to Test Protective Effects of Food Antioxidant Compounds

**DOI:** 10.3390/molecules26195786

**Published:** 2021-09-24

**Authors:** Cristina Arteaga, Nuria Boix, Elisabet Teixido, Fernanda Marizande, Santiago Cadena, Alberto Bustillos

**Affiliations:** 1 Toxicology Unit, Pharmacology, Toxicology and Therapeutical Chemistry Department, Pharmacy School, University of Barcelona, Avda Joan XXIII s/n, 08028 Barcelona, Spain; ca.arteaga@uta.edu.ec (C.A.); nuriaboix@ub.edu (N.B.); eteixido1511@ub.edu (E.T.); 2 Faculty of Health Sciences, Nutrition and Dietetics, Technical University of Ambato, Ambato 180207, Ecuador; mf.marizande@uta.edu.ec; 3 INSA-UB Nutrition and Food Safety Research Institute, Food and Nutrition Torribera Campus, University of Barcelona, Prat de la Riba 171, 08921 Santa Coloma de Gramenet, Spain; 4 Faculty of Applied Sciences, International SEK University, Quito 170134, Ecuador; sacadena.mbme@uisek.edu.ec; 5 Faculty of Health Sciences, Medicine, Technical University of Ambato, Ambato 180207, Ecuador

**Keywords:** oxidative stress, zebrafish embryo, antioxidant effect, polyphenols, carotenoids

## Abstract

The antioxidant activity of food compounds is one of the properties generating the most interest, due to its health benefits and correlation with the prevention of chronic disease. This activity is usually measured using in vitro assays, which cannot predict in vivo effects or mechanisms of action. The objective of this study was to evaluate the in vivo protective effects of six phenolic compounds (naringenin, apigenin, rutin, oleuropein, chlorogenic acid, and curcumin) and three carotenoids (lycopene B, β-carotene, and astaxanthin) naturally present in foods using a zebrafish embryo model. The zebrafish embryo was pretreated with each of the nine antioxidant compounds and then exposed to tert-butyl hydroperoxide (tBOOH), a known inducer of oxidative stress in zebrafish. Significant differences were determined by comparing the concentration-response of the tBOOH induced lethality and dysmorphogenesis against the pretreated embryos with the antioxidant compounds. A protective effect of each compound, except β-carotene, against oxidative-stress-induced lethality was found. Furthermore, apigenin, rutin, and curcumin also showed protective effects against dysmorphogenesis. On the other hand, β-carotene exhibited increased lethality and dysmorphogenesis compared to the tBOOH treatment alone.

## 1. Introduction

Reactive oxygen species (ROS) and reactive nitrogen species (RNS) are originated during cell metabolism. They are essential to a normal physiological state, but they participate in pathological processes when in excess [1]. Aerobic organisms have defenses to prevent ROS-induced oxidative damage, involving antioxidant enzymes and/or non-enzymatic mechanisms, including endogenously produced antioxidant compounds or the ingestion of antioxidants in the diet [2].

The imbalance, when the concentration of reactive species is higher than the antioxidant defenses of the organism, is called oxidative stress (OS) [3]. The consequences of OS include macrophage recruitment; the inhibition of the normal functioning of lipids and proteins; and mitochondrial, membrane, and DNA damage [4,5,6,7]. These alterations have been correlated with several pathologies, such as cancer, aging, diabetes, rheumatoid arthritis, and cardiovascular and neurodegenerative diseases, among others [8,9,10,11].

Several studies have found that an organism requires the intake of antioxidants through the diet to reduce oxidative damage [12] in physiopathological situations (due to UV exposure, smoking, polluted air, etc.), which produce excess ROS. Various antioxidants are ingested through the diet, such as phenolic compounds, vitamins, carotenoids, and flavonoids. The term “phenolic compounds” refers to any substance with a phenol group attached to aromatic or aliphatic structures. Phenolic compounds come from plants and are among the most important secondary metabolites; their presence in the animal kingdom is due to their consumption through the diet. Among these compounds, flavonoids are the most studied and abundant; their chemical structures contain a flavonic nucleus that consists of 15 carbon atoms arranged in three rings (C6–C3–C6) [13]. Their antioxidant mechanisms include the inhibition of enzymes or chelation of trace elements involved in producing free radicals, the uptake of ROS, and the protection of endogenous antioxidant defenses [14]. The mean flavonoid intake is estimated at 23 mg/day [15,16], and the primary sources are black tea, red wine, onions, apples, and beer [17,18].

Another group of compounds that have been studied because of their antioxidant activity are the carotenoids. They are pigments whose structures comprise a series of conjugated C = C bonds (polyene), which allow them to interact with free radicals; therefore, they can act as effective antioxidants [19]. Carotenoids are broadly distributed in natural systems, and their role in preventing different diseases has been studied, mainly for the compounds present in the diet such as β-carotene, lutein, and lycopene [18,20,21].

Identifying the roles of antioxidants in diseases and disorders correlated with oxidative processes is essential for analyzing protective effects in vivo. For this reason, our laboratory has developed a zebrafish (ZF) embryo model for evaluating the protective effects of these antioxidant compounds [22]. Oxidative stress is induced using tert-butyl hydroperoxide (tBOOH). tBOOH generates butoxyl radicals through Fenton’s reaction [23]. The radicals formed favor the intracellular depletion of thiol groups and glutathione reserves, producing a significant increase in lethality and dysmorphogenesis in exposed zebrafish embryos. This model allows a comparison between the concentration–effect curves of lethality and dysmorphogenesis for zebrafish embryos exposed to tBOOH and the curves of embryos pretreated with antioxidants; statistical analysis can be performed to explore the protective effect of the analyzed antioxidant compound.

The objective of the present work was to evaluate the in vivo protective effects of food compounds with antioxidant activity against oxidant-induced developmental toxicity in zebrafish embryos.

## 2. Results

### 2.1. Concentration Effect Curves for tBOOH in Zebrafish Embryos

Our research group previously developed and validated a ZF embryo oxidative stress model with which to evaluate the protective activity of antioxidant substances (22).

**Figure 1:**
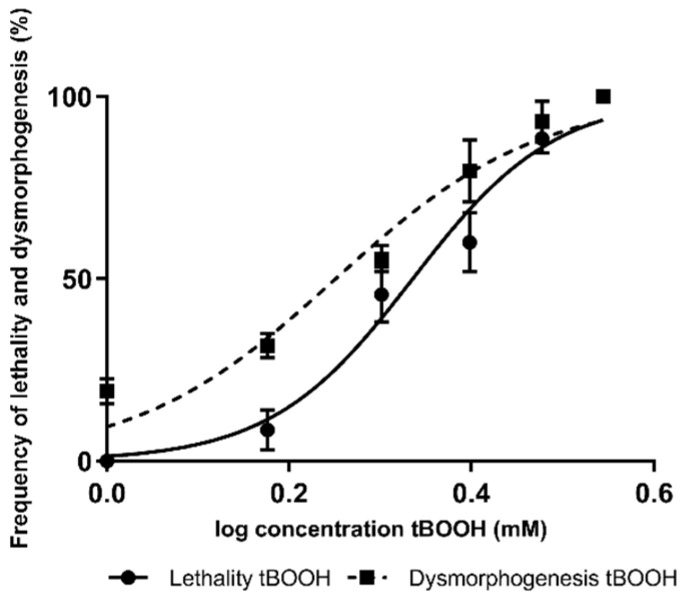
Concentration–response curves for lethality and dysmorphogenesis produced using tert-butyl hydroperoxide (tBOOH).

Zebrafish embryos are exposed to tert-butyl hydroperoxide (tBOOH) to obtain lethality and dysmorphogenesis curves. The zebrafish embryos are exposed to tBOOH 24 to 48 h post-fertilization (hpf) at different concentrations, ranging from 1 to 3.5 mM (Figure 1). The lethal concentration 50 (LC_50_) was discovered to be 2.1 mM, whereas the effective concentration 50 for dysmorphogenesis (EC_50_) was 1.7 mM. The curves mentioned above were used to compare zebrafish embryos previously exposed, or not, to antioxidant compounds, after which they were exposed to tBOOH.

### 2.2. Identification of the Protective Effects of Antioxidant Compounds in Zebrafish Embryos

The previously described zebrafish model was used to evaluate the protective effects of six polyphenols and three carotenoids present in food.

**Figure 2:**
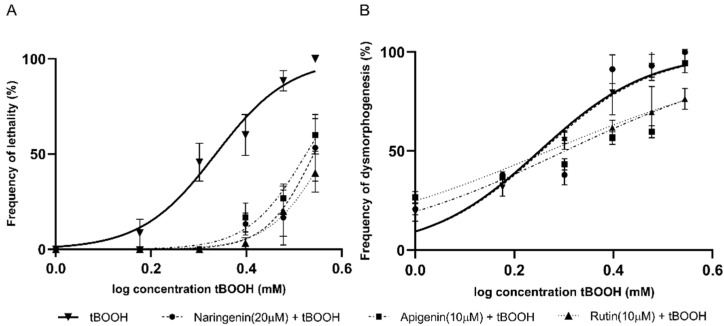
Concentration–response curves produced by tBOOH, in combination with different flavonoid compounds for (**A**) lethality and (**B**) dysmorphogenesis.

Out of the six polyphenols, three were flavonoids: naringenin (20 µM), apigenin (10 µM), and rutin (10 µM). The three flavonoids produced a significant drift in the concentration–response curves for lethality. Furthermore, apigenin and rutin showed protective effects against dysmorphogenesis, whereas naringenin did not exhibit any protective effect against dysmorphogenesis (Figure 2).

**Figure 3:**
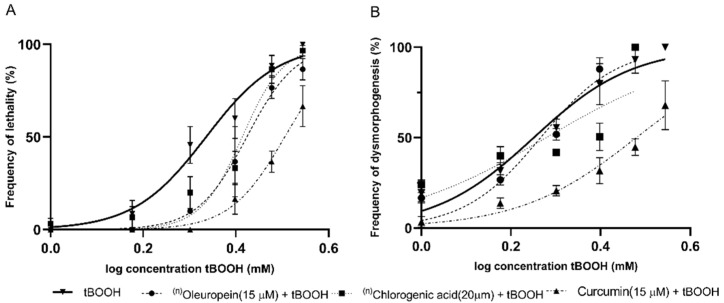
Concentration–response curves produced using tBOOH in combination with different polyphenolic compounds for (**A**) lethality, and (**B**) dysmorphogenesis. ^(n)^Note: the absence of points on the graph at higher concentrations is because they already produced a 100% embryo lethality in all replicates.

Oleuropein (15 µM), chlorogenic acid (20 µM), and curcumin (15 µM) were also analyzed. These polyphenols resulted in a significant drift in the concentration–response curves for lethality. Only curcumin exhibited a protective effect against dysmorphogenesis (Figure 3).

**Figure 4:**
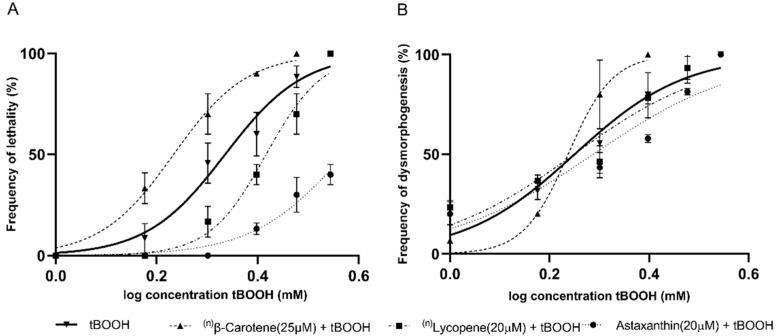
Concentration–response curves produced using tBOOH in combination with different carotenoid compounds for (**A**) lethality, and (**B**) dysmorphogenesis. ^(n)^Note: The absence of points on the graph at higher concentrations is because they already produced a 100% embryo lethality in all replicates.

In addition, the carotenoids lycopene (20 µM), astaxanthin (20 µM), and β-carotene (25 µM) were evaluated. Lycopene and astaxanthin resulted in a significant drift in the concentration–response curves for lethality. By contrast, none of the carotenoids showed a protective effect against dysmorphogenesis (Figure 4). Furthermore, β-carotene resulted in a leftward drift in the curves for lethality and dysmorphogenesis, which could indicate a possible prooxidant effect.

Table 1 presents the flavonoid and carotenoid compounds used, showing the LC_50_ and values; eight evaluated compounds exhibited protective effects against lethality after the oxidant treatment. However, for the EC_50_ values of dysmorphogenesis, only rutin, apigenin, and curcumin showed protective effects. On the other hand, β-carotene (25 µM) exhibited an increased risk of mortality and dysmorphogenesis in zebrafish after the oxidant treatment, based on a significant reduction in the LC_50_ (2.6 mM) and EC_50_ (1.5 mM) values.

## 3. Discussion

Oxygen is essential for human life; however, at the same time, it produces toxic substances, such as free radicals and reactive oxygen species (ROS); these substances are oxidizing, unstable, and reactive. Furthermore, they can react with any macromolecule and cause cell damage [24]. To counteract these oxidizing substances, the body employs antioxidant enzymes, such as superoxide dismutase and glutathione peroxidase, and antioxidant compounds derived from the diet. Therefore, the study of the antioxidant capacity of compounds has been garnering interest in the past few years. There are several in vitro techniques for determining antioxidant activity, although they have limitations from a nutritional point of view because none reproduces a physiological situation [25]. For this reason, a method that included in vivo techniques would lead to more impactful results, because oxidative stress implies mechanisms that depend on many system conditions, especially the kinetic parts of reactions. Our team used a ZF embryo model [22], which could be a valuable in vivo method, to test the protective effects of nine antioxidant compounds that have been broadly studied in vitro. We evaluated six phenolic compounds and three carotenoids. Phenolic compounds represent an important contribution to the antioxidizing potential of the human diet; of these compounds, flavonoids are the most studied and abundant. The antioxidant activity of the flavonoids apigenin, rutin, and naringenin was studied. These flavonoids are bioactive compounds mainly found in various fruits, plants and vegetables, nuts, and onions. In vitro studies have shown that these flavonoids effectively neutralize hydroxyl radicals, superoxide, hydrogen peroxide, nitric oxide radicals, DPPH, and lipid peroxidation [26,27,28,29]. Chen et al., in 2012 [30], performed a QSAR analysis using a zebrafish larva model to evaluate the ROS-scavenging capacities of fifteen flavonoids, including rutin, against UV-induced phototoxicity. In accordance with previous studies, they concluded the importance of the two hydroxyl groups and their positions, with at least two hydroxyl groups necessary for a strong biological activity [30,31]. Furthermore, it was determined that hydroxyl groups at positions C3, C5, and C7 confer better flavone stability and activity [31]. Our results showed a protective effect against tBOOH-induced lethality for the three flavonoids. Apigenin and rutin also showed protective effects against dysmorphogenesis; however, naringenin did not show any effect against dysmorphogenesis.

In addition to those of flavonoids, the antioxidant effects of oleuropein, chlorogenic acid, and curcumin were also evaluated. In vitro and in vivo studies have shown that these three phenolic compounds have important antioxidant effects [32,33,34]. Oleuropein is a biophenol found in olive leaves, extra virgin olive oil, and some species of the Oleaceae family [32]. Chlorogenic acids (CGAs) are esters formed between caffeic and quinic acids and represent a group of polyphenols present in the human diet [35]. Several studies have shown that drinking beverages containing CGAs such as coffee, tea, wine, and various fruit juices reduces the risk of developing different chronic diseases [36,37,38]. One of the reasons for this reduction is the antioxidant capacity of the CGAs, which donate hydrogen atoms to reduce free radicals and inhibit oxidation reactions [35]. Curcumin is a polyphenol that is used for coloring and seasoning in food products. Its antioxidant activity has been studied during the last few years, and one study suggests that it can protect biomembranes against peroxidative damage [39]. Using the ZF embryo model, it was observed that pretreatment with oleuropein, chlorogenic acid, or curcumin reduced the mortality-inducing effect of tBOOH-induced oxidative stress; however, a significant protective effect against dysmorphogenesis was only observed for curcumin.

Another group with antioxidant properties is the carotenoids, a ubiquitous group of isoprenoid pigments. They are quenchers of singlet oxygen and scavengers of ROS [40]. The molecular mechanisms underlying the anti- and pro-oxidant activity of carotenoids are still not fully understood. Among the most studied carotenoids are lycopene and β-carotene. These can be found abundantly in tomatoes, tomato sauce, various fruits, algae, and vegetables [18,41]. When evaluating the protective effects of these carotenes, it was found that lycopene showed antioxidant activity, with a protective effect against embryonic lethality; however, no effect against dysmorphogenesis was found. On the other hand, β-carotene increased the incidence of lethality and dysmorphogenesis in the ZF embryos compared to the effect of the oxidant alone; this is in accordance with studies that have shown that high doses of β-carotene have antioxidant effects that are followed by a prooxidant action at high oxygen tension, which may be related to its adverse effects [42]. Moreover, a study showed that β-carotene supplementation had no protective effect on the total mortality of diabetic male smokers compared with a placebo [43]. Another carotenoid evaluated was astaxanthin; it is a xanthophyll carotenoid found in algae, yeast, salmon, trout, krill, shrimp, and crayfish. It is a red, fat-soluble antioxidant pigment that has no pro-Vitamin A activity [44]. In our study, astaxanthin exhibited a protective effect against lethality, but no effect against dysmorphogenesis was found.

In conclusion, eight of the nine molecules evaluated showed antioxidant activity with protective effects against ZF embryonic lethality. Only apigenin (10 µM), rutin (10 µM), and curcumin (15 µM) additionally exhibited protective effects against dysmorphogenesis resulting from tBOOH-induced oxidative stress. By contrast, it was found that β-carotene produced the opposite effect, increasing the mortality and dysmorphogenesis rate, because it reduced the LC_50_ and EC_50_ values. The balance and timing of oxidative and antioxidative forces is key to the proper regulation and timing of embryonic development [45]. Differences in the kinetics or mechanism of action of these antioxidants could be the leading reason for the different protective capacities against dysmorphogenesis. More studies are needed to explore why only some compounds showed protective effects on morphogenesis during embryonic development. This study tried to discriminate between embryotoxic effect (lethality) and dysmorphogenic effect (teratogenicity). In some cases, malformations are likely to precede and result in death. In other instances, lethality and malformation may be due to different causes. The independence of these two manifestations would be suspected if a compound increased the separation between the lethal and dysmorphogenic concentration–response curves. All the antioxidant compounds tested in our study did not increase the teratogenic effect over the lethal dose more than twice; therefore, no increased teratogenic potential was observed for any antioxidant compound [46].

Altogether, these results indicate that this ZF embryo model is a valuable tool with which to analyze the protective effects of the antioxidant molecules that constitute food. To determine the chemical–structural reasons for which apigenin, rutin, and curcumin showed the highest protective effects in our study, further analyses are necessary; for instance, to determine quantitative structure–activity relationships (QSARs).

## 4. Materials and Methods

### 4.1. Ethical Statement

The procedures involving zebrafish larvae and embryos were authorized by the Animal Ethics Committee of the University of Barcelona, authorization number or protocol 7971 of the Department of Livestock and Fisheries of the Government of Catalonia (Procedure DAAM 7971).

### 4.2. Chemicals and Solution Preparation

Tert-butyl hydroperoxide (tBOOH, CAS number: 75-91-2) and the antioxidant compounds were acquired from TCI Europe. tBOOH was dissolved in 0.3X Danieau’s buffer (17.4 mM NaCl; 0.23 mM KCl; 0.12 mM MgSO_4_·7 H2O; 0.18 mM Ca(NO_3_)_2_; 1.5 mM HEPES (N-(2-hydroxyethyl) piperazine-N0 -(2-ethanesulfonic acid); pH 7.4).

Naringenin (20 μM) (CAS number: 67604-48-2), oleuropein (15 μM) (CAS number: 32619-42-4), rutin (10 μM) (CAS number: 207671-50-9), chlorogenic acid (20 μM) (CAS number: 327-97-9), apigenin (10 μM) (CAS number: 520-36-5), curcumin (15 μM) (CAS number: 458-37-7), lycopene (20 μM) (CAS number: 502-65-8), astaxanthin (20 μM) (CAS number: 472-61-7), and β-carotene (25 μM) (CAS number: 7235-40-7) were acquired from Sigma-Aldrich^®^. Antioxidants were dissolved in 100% dimethyl sulfoxide (DMSO, Sigma-Aldrich, Madrid, Spain) and subsequently diluted in 0.3× Danieau’s buffer to a final DMSO concentration of 0.05% (*v*/*v*). Antioxidants were used at different concentrations, depending on the highest concentration at which no effect on lethality or embryonic development was observed (maximum tolerable concentration, MTC)

### 4.3. Zebrafish Maintenance and Egg Production

Adult wild-type zebrafish were housed in standardized conditions. The embryos were collected, cleaned, and selected according to their viability. The fertilized embryos were treated with water standardized according to the International Organization for Standardization (ISO) standards 7346-1 and 7346-2 (ISO, 1998; 2 mM CaCl_2_·2H_2_O, 0.5 mM MgSO_4_·7H_2_O, 0.75 mM NaHCO_3_, and 0.07 mM KCl). Fertilized eggs were staged according to previous studies by Kimmel et al., 1995 [47], and selected for subsequent exposure under a dissecting stereomicroscope (Motic SMZ168, Motic China Group, LTD., Luwan, Shanghai, China). The fish embryos were kept in glass vials at a controlled temperature of 27 ± 1 °C.

### 4.4. Exposure of Zebrafish Embryos to Oxidative Stress (Tert-Butyl Hydroperoxide)

**Figure 5:**
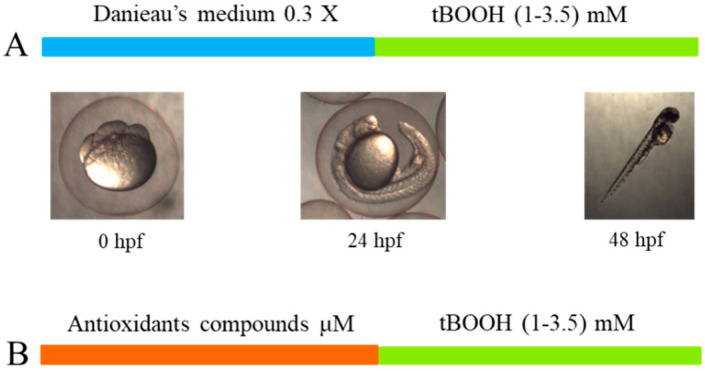
(**A**) Schematic overview of the process for obtaining the lethality and dysmorphogenesis curves for tBOOH. (**B**) Process for evaluating the protective effects of the antioxidant compounds.

For the preparation of the tBOOH curve, the methodology of Boix, 2020, was followed. This methodology is based on obtaining a concentration–lethality response (LC_50_) curve and dysmorphogenesis (EC_50_) by exposing the ZF embryos to an oxidative stress inducer, tert-butylhydroperoxide (tBOOH). Once zebrafish embryos are obtained, they are kept in 0.3X Danieau’s medium from 0 to 24 hpf. From 24 to 48 hpf, the embryos are exposed to tBOOH solutions at different concentrations, of 1, 1.5, 2, 2.5, 3, and 3.5 mM. Embryos from 3 different clutches of zebrafish in triplicate were used (Figure 5A).

### 4.5. Determination of the Protective Effects of Antioxidant Compounds

To establish whether a compound had a protective effect against oxidative stress, zebrafish embryos were first exposed to the antioxidant compound from 0 to 24 hpf. The concentrations were calculated depending on the maximum tolerable concentration assays. Then, from 24 to 48 hpf, the embryos were exposed to the stress inducer, tBOOH. Subsequently, each group of embryos was evaluated at each of the concentrations of tBOOH (Figure 5B). To determine whether there was a significant difference, the curve for exposure to tBOOH alone and the curve for pre-exposure to the antioxidant compound were compared.

Ten fertilized eggs were exposed to 2.5 mL for each substance and concentration. Three independent replications were performed, using eggs from different spawning events. The ZF embryos were pre-exposed to the antioxidant compounds for 24 h, then the antioxidant solution was removed, washing was performed using Danieau’s medium, and the ZF embryos were exposed to different tBOOH concentrations. The lethality was evaluated after 48 h, and the mean of dead embryos was calculated after the appropriate assays. For the dysmorphogenesis evaluation, we followed the scoring system described by Teixidó et al. [48] to compute the embryos’ dysmorphogenesis at around 48 hpf. We selected nine morphological features, described in Table 2. The frequency of abnormal embryos was calculated (defined as the embryos with a score 1 in any morphological feature) for each concentration and treated group.

A shift of the concentration–response curve to the right due to pre-exposure to the antioxidants indicates a protective effect against the oxidative stress inducer, because a higher concentration of inducer is required to obtain the same results as those with the exposure to tBOOH alone. Due to the pre-exposure to antioxidants, a shift to the left of the concentration–response curve implies an increase in oxidative stress.

### 4.6. Statistical Analysis

The concentration–response curves for mortality and dysmorphogenesis were calculated and evaluated using GraphPad 7.02 Software Inc. The extra-sum-of-squares F test was used to compare the fit of the parameters of each data group of the curve. The confidence interval was adjusted to 95%.

## 5. Conclusions

This study used zebrafish embryos as a model organism to test the protective capacity of six phenolic compounds and three carotenoids commonly found in foods. All the compounds, except β-carotene, showed protective effects against oxidative-stress-induced lethality. Furthermore, apigenin, rutin, and curcumin also exhibited protective effects against tBOOH-induced dysmorphogenesis. We propose that a zebrafish embryo test, as presented here, could be applied to evaluate the in vivo protective effects of novel bioactive food components with potential antioxidant capacity.

## Figures and Tables

**Table 1:** Effects of polyphenols and carotenoid compounds against an oxidant inducer (tBOOH) of developmental toxicity in zebrafish.

Compound	Lethality				Dysmorphogenesis			
	LC_50_ (mM) tBOOH (95% CI)	LC_50_ (mM) (Compound + tBOOH) (95% CI)	*p*-Value	Effect	EC_50_ (mM) tBOOH (95% CI)	EC_50_ (mM) (Compound + tBOOH) (95% CI)	*p*-Value	Effect
Naringenin (20 μM)	2.1 (2.0–2.2)	3.4 (3.3–3.7)	<0.0001	PE ^1^	1.7 (1.6–1.8)	1.8 (1.5–2.0)	>0.05	S/E ^2^
Apigenin (10 μM)	2.1 (2.0–2.2)	3.3 (3.2–3.4)	<0.0001	PE	1.7 (1.6–1.8)	2.0 (1.7–2.2)	0.0006	PE
Rutin (10 μM)	2.1 (2.0–2.2)	3.6 (3.5–3.9)	<0.0001	PE	1.7 (1.6–1.8)	1.8 (1.7–2.0)	<0.0008	PE
Oleuropein (15 μM)	2.1 (2.0–2.2)	2.6 (2.5–2.7)	<0.0001	PE	1.7 (1.6–1.8)	1.8 (1.7–1.9)	>0.05	S/E
Chlorogenic acid (20 μM)	2.1 (2.0–2.2)	2.5 (2.4–2.6)	0.0028	PE	1.7 (1.6–1.8)	1.8 (1.5–2.2)	>0.05	S/E
Curcumin (15 μM)	2.1 (2.0–2.2)	3.2 (3.1–3.2)	<0.0001	PE	1.7 (1.6–1.8)	3.0 (2.8–3.2)	<0.0001	PE
Lycopene (20 μM)	2.1 (2.0–2.2)	2.6 (2.5–2.6)	0.0003	PE	1.7 (1.6–1.8)	1.7 (1.5–1.9)	0.56	S/E
β-carotene (25 μM)	2.1 (2.0–2.2)	1.7 (1.6–1.7)	0.0001	IL ^3^	1.7 (1.6–1.8)	1.5 (1.5–1.6)	0.01	ID ^4^
Astaxanthin (20 μM)	2.1 (2.0–2.2)	3.7 (3.5–3.9)	<0.0001	PE	1.7 (1.6–1.8)	1.9 (1.7–2.1)	0.0506	S/E

^1^ PE: protective effect. ^2^ S/E: no effect because there is no difference regarding the 95% confidence interval. ^3^ IL: increased lethality. ^4^ ID: increased dysmorphogenesis. Values of LC_50_ and EC_50_ are expressed in mM.

**Table 2:** Criteria employed to evaluate dysmorphogenesis on zebrafish embryos.

Morphological Features	Morphological Abnormality	Example of Observed Characteristics.
Detachment of the tail; Tail	No tail, malformation of chorda or spinal cord. Tail necrosis, bent tail.	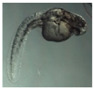
Optic system; Otic system; Brain	Abnormal pigmentation, asymmetric eyes. Formation of no, one, or more than two otoliths per sacculus. Brain necrosis, hemorrhage.	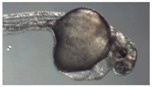
Heart	Pericardial edema, big heart, hemorrhage, abnormal chambers.	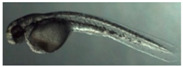
Head-body pigmentation; Tail pigmentation	Lack of pigmentation in the tail or body.	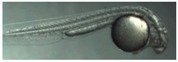
Movement	Spasms, abnormal movements, no movement at all.	The ZF embryo was touched and its movement was evaluated.
* Control	No signs of dysmorphogenesis.	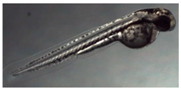

* Control of normal characteristics of a zebrafish embryo at 48 hpf.

## Data Availability

All the data are included on this paper.
